# Institutional barriers and enablers to implementing and complying with internationally accepted quality standards in the local pharmaceutical industry of Pakistan: a qualitative study

**DOI:** 10.1093/heapol/czz054

**Published:** 2019-07-13

**Authors:** Fatima Tauqeer, Kirsten Myhr, Unni Gopinathan

**Affiliations:** 1 Oslo Group on Global Health Policy, Department of Community Medicine and Global Health, Faculty of Medicine, Institute of Health and Society, University of Oslo, PO Box 1130 Blindern, Oslo, Norway; 2 Centre for Global Health, Faculty of Medicine, University of Oslo, PO Box 1130 Blindern, Oslo, Norway; 3 Department of Community Medicine and Global Health, Institute of Health and Society, University of Oslo, PO Box 1130 Blindern, Oslo, Norway; 4 Cluster for Global Health, Division for Health Services, Norwegian Institute of Public Health, PO Box 222 Skøyen, N-0213 Oslo, Norway

**Keywords:** Quality of medicines, medicines quality assurance, local pharmaceutical production, good manufacturing practices

## Abstract

Complying with good manufacturing practices (GMP) and ensuring a quality system is integral to production and supply of quality medicines and achieving universal health coverage. This study focus on the local production of medicines in Pakistan, a lower middle-income country that has observed considerable growth in the number of pharmaceutical companies over the past two decades. Against this background, we investigated: (1) How is quality assurance (QA) and GMP compliance understood and acted upon by local pharmaceutical manufacturers?; (2) What are the institutional barriers and enablers for QA and GMP compliance in the local pharmaceutical sector from the perspective of key stakeholders?; and (3) What are the institutional barriers and enablers for strengthening local regulatory capacity to improve QA in the industry in the long term? We used a qualitative study design involving 22 interviews of the drug regulatory bodies (*n* = 9), academia (*n* = 3) and local manufacturers (*n* = 10), identifying key themes in data by thematic analysis. Document analysis was used to collect additional information and supplement the interview data. We identified that manufacturing facilities operated under different GMP standards and interpretations, pointing towards an absence of harmonization in quality standards across the industry. Views diverged about the status of GMP compliance, with interviewees from academia presenting a more critical view compared with regulators who promoted a more positive story. Among the barriers explaining why companies struggled with quality standards, the lack of a mindset promoting quality and safety among profit-oriented manufacturers was prominent. At the federal level, DRAP’s establishment represented an institutional improvement aiming to promote QA through inspections and guidance. While some positive measures to promote quality have been observed, the need for DRAP to strengthen its technical and regulatory capacity, enhance its engagement in international collaboration and learning, and improve transparency and accountability were highlighted. Overall, since the challenges in Pakistan are shared with other low- and middle-income countries with local production, there is a need to commit to international collaborative mechanisms, such as those lead by WHO, on this issue.

**Key Messages**

- Local production and regulation of medicines in Pakistan, a lower middle income country, face a set of challenges, despite recent institutional improvements, in following internationally accepted quality standards for production of medicines.
- National regulatory bodies such as DRAP together with other public health institutions play a crucial role in holding pharmaceutical companies accountable for ensuring the safety of their products when bringing these to markets.
- National regulatory authorities in low- and middle-income countries need to commit to international collaborative mechanisms, such as those lead by WHO, to enhance their technical capacity to safeguard availability of quality medicines. This would require increased public investment and strengthening of its own institutional capacity building.
- A synergy between the pharmaceutical industry and the regulatory body to promote information-sharing can play a strong role in encouraging, developing and training in quality assurance, incorporating good manufacturing practices.

## Introduction

Access to essential medicines is one of the six building blocks of the World Health Organisation’s (WHO) framework for health systems (WHO, 2010) and universal health coverage (UHC) is a major target of the sustainable development goal 3 on health (UNDESA, 2018). On the path to UHC, it is crucial for governments worldwide to provide a continuous supply of quality medicines at affordable prices. Globally, the problem of substandard and falsified (SF) medicines is associated with morbidity and mortality, particularly in low- and middle-income countries (LMICs) (Newton *et al.*, 2010; Bassat *et al.*, 2016; Petersen *et al.*, 2017; WHO, 2018b, 2018d). WHO defines substandard (also called out of specification) products as ‘authorized medicinal products that fail to meet either their quality standards or specifications, or both’ (WHO, 2018b).

The WHO framework for health systems and the WHO Medicines Quality Assurance Programme recommend that pharmaceutical manufacturers comply with quality assurance (QA) guidelines including good manufacturing practices (GMP) (WHO Drug Information, 2017). QA of medicines is a broad mechanism that guards patient safety by taking care of an extensive range of processes that affect the quality of medicines (WHO, 2018c). The GMP guidelines cover all manufacturing processes such as quality management, sanitation, hygiene, qualification, validation, complaints, product recalls, self-inspection, personnel, premises, equipment, materials, documentation, production, quality control and more (WHO, 2014a; WHO Drug Information, 2017). Adherence to GMP is the first step towards ensuring that pharmaceutical products are manufactured and controlled to meet the quality standards set out by the regulatory authorities (Institute of Medicine, 2013). Adherence to these standards is vital to reduce risk exposure such as cross-contamination and false labelling, and to supply quality medicines to patients (Patel and Chotai, 2011; Geyer *et al.*, 2018).

Globally, different sets of GMP guidelines or legal standards are available, e.g. by WHO (WHO Drug Information, 2017), USFDA (U.S. Food and Drug Administration, 2018), EC (European Commission, 2018) and ICH (International Conference on Harmonization, 2019). Yet in many LMICs, these standards have not been met by adequate levels of regulatory capacity to enforce them, and their implementation fails to keep pace (Johnston and Holt, 2014; Brhlikova *et al.*, 2015; Pezzola and Sweet, 2016; Roth *et al.*, 2018). Non-adherence to GMP is a fundamental challenge contributing to the production of substandard medicines, risking adverse public health effects (Chin and Lee, 2008; Institute of Medicine, 2013; Johnston and Holt, 2014; WHO, 2018b). In the current environment of globalized production of pharmaceuticals, harmonization of pharmaceutical standards is a global challenge (Pezzola and Sweet, 2016). However, WHO’s regulatory systems strengthening team is envisioning a gradual move towards regulatory convergence and harmonization by collaborating with national regulatory bodies (Azatyan, 2017).

Pakistan is among several LMICs that have experienced considerable growth of their local pharmaceutical industry (Aamir and Zaman, 2011). The manufacturing facilities produce generics and branded generics, meeting 70–80% of the country needs (Aamir and Zaman, 2011; Atif *et al.*, 2017; Policy Research Institute of Market Economy, 2017). Previously, studies on drug quality in Pakistan have identified cases where antibiotics have lacked the correct amount of active ingredient and had traces of impurities (Johnston and Holt, 2014), production errors related to antimalarials (Johnston and Holt, 2014), and samples of active ibuprofen ingredient indicating low compliance with standard assay values (Babar *et al.*, 2016).

In spite of the growing number of manufacturers, there is a shortage of literature on major barriers and opportunities to implementing GMP standards and QA in manufacturing facilities in LMICs. There is particularly a lack of research investigating how QA and GMP compliance is understood, experienced and acted upon. Moreover, the key challenges faced by local regulatory institutions when assessing whether locally manufactured products meet internationally agreed quality standards and their perspectives on industry compliance remain poorly characterized. Against this background, three main research questions were formulated for this study: (1) How is QA and GMP compliance understood and acted upon by local pharmaceutical manufacturers?; (2) What are the institutional barriers and enablers for QA and GMP compliance in the local pharmaceutical sector of Pakistan from the perspective of key stakeholders?; and (3) What are the institutional barriers and enablers for strengthening local regulatory capacity to improve QA in the long term?

## Materials and methods

### Study design

This study adopted a qualitative case study design involving semi-structured interviews and document review. The case in focus was QA and GMP implementation among local pharmaceutical manufacturers, investigated from the experience and perspectives of individuals from local pharmaceutical companies, drug regulatory authorities and academia. To report on the characteristics of the research team, study design and data analysis, we used the COnsolidated criteria for REporting Qualitative research (COREQ) (Supplementary File S1). COREQ enables transparent reporting on various choices important for understanding the study design and the collection and interpretation of qualitative data.

### Study setting

This study took place in Pakistan. The main study site was Lahore, where interviews with individuals from local manufacturing facilities and regulators were conducted. In addition, regulators and academics with relevant experience and knowledge were also recruited from Islamabad and Karachi. Data were collected from November 2017 to February 2018.

The pharmaceutical sector of Pakistan is ranked 10th largest in the Asia-Pacific region (Atif *et al.*, 2017). In 2019, the total number of registered pharmaceutical companies was 647 (DRAP Press Release, 2019). The landscape has changed from most companies being multinational companies (MNCs) in the 1990s to the current situation where only 20–30 are MNCs (Aamir and Zaman, 2011; Policy Research Institute of Market Economy, 2017). Previously, MNCs dominated the market shares, but recent estimates suggest that local companies meet the majority of the national demand (Atif *et al.*, 2017; Policy Research Institute of Market Economy, 2017), in part due to some MNCs leaving Pakistan (Aamir and Zaman, 2011; Rashid, 2015; Policy Research Institute of Market Economy, 2017). The total size of the pharmaceutical market is estimated to be between 1.6 and $3.5 billion (Aamir and Zaman, 2011; Rashid, 2015; Policy Research Institute of Market Economy, 2017). In comparison, the total global pharmaceutical market has since 2014 exceeded $1 trillion (Statista, 2019), which means that Pakistan’s pharmaceutical market is around 0.5% of the global market. Approximately 50 000 drugs and 1100–1200 drug molecules (active pharmaceutical ingredients, APIs) are registered (Atif *et al.*, 2017). The industry’s own assessment shows that the top 50 companies have 89% and the top 100 companies 97% of the market shares, leaving the rest to compete for a low share of the domestic market (Policy Research Institute of Market Economy, 2017). One explanation for the growth of local companies, despite the low market shares, has been that some companies produce for neighbouring markets (e.g. companies near the border of Afghanistan) (Policy Research Institute of Market Economy, 2017). Other factors explaining the growth of the local pharmaceutical industry, such as public-sector support, were not covered by the literature we reviewed.

The Drug Regulatory Authority of Pakistan (DRAP), established under the DRAP Act 2012, is under the administrative control of the Federal Health Ministry. DRAP is an autonomous body responsible for the enforcement of pharmaceutical regulation in Pakistan under the Drugs Act of 1976 (DRAP, 2019a). It is responsible for regulating the manufacturing, import, export, storage, distribution and sale of medical products (DRAP, 2019a). In addition, Provisional Quality Control Boards (PQCB), established in the 1980s under the same act, regulate market surveillance of registered medical products (Atif *et al.*, 2017). DRAP was established as a result of a tragic incident in 2012 that took >200 lives due to the administration of a substandard antihypertensive drug at the Punjab Institute of Cardiology, in Lahore. Investigational reports revealed that the medicine was contaminated with the antimalarial pyrimethamine due to a manufacturing error (Chaudhry, 2013; WHO, 2013a, 2018b). Before the establishment of DRAP and its regional offices, drug regulation was under the Ministry of Health (MOH) through a federal medicines regulatory authority, which policy experts argued was performing poorly (Nishtar and Mehboob, 2011). After the 18th constitutional amendment in 2011, the MOH was dissolved and governance of healthcare systems, including drug regulation, was regionalized to the five provinces (Atif *et al.*, 2017). Later, the need to establish a federal regulatory body was put forward by the pharmaceutical industry and public health proponents (Nishtar, 2013; Rashid, 2015). However, the drug regulation landscape, even after the establishment of DRAP in 2012, has according to scholars, remained ineffective and runs in an intricate social, political, institutional and cultural context (Nishtar, 2013; Rashid, 2015; GARP – Pakistan, 2018).

### Theoretical perspectives

The theoretical underpinnings for this study were gained from reviewing the literature to clarify factors that might explain challenges to QA and GMP compliance and the prevalence of substandard medicines in LMICs, including the political economy of the context in which GMP is implemented. Previously, WHO has reported that factors like inadequate QA during drug manufacturing, the absence or inability of drug regulatory authorities, an abundance of small pharmaceutical producers that overload existing drug regulatory capacity, and high prices and inefficient collaboration among stakeholders lead to production and supply of substandard medicines (WHO, 1999). Additionally, factors like non-adherence or inadequate adherence to GMP have repeatedly been associated with producing substandard medicines (Caudron *et al.*, 2008; Institute of Medicine, 2013; Kelesidis and Falagas, 2015; Nwokike *et al.*, 2018). Finally, the WHO Global Surveillance and Monitoring System for SF medical products highlights limited access to medicines, poor governance and insufficient technical capacity as factors underlying the rise of SF medical products (WHO, 2018b). These pre-identified factors and explanations formed the basis on which we identified the main research questions. Moreover, the knowledge gained from the literature guided us when reviewing policy documents and conducting semi-structured interviews and facilitated our interpretation of the qualitative data.

### Study sample

We purposively (Patton, 2002) recruited stakeholders from three different groups of the pharmaceutical sector, who collectively could provide a diverse range of experiences and perspectives to answer the research questions. We defined these stakeholders as follows:

1. the regulatory group, comprised of interviewees from DRAP, drug testing laboratory and PQCB;
2. the manufacturing group, comprised of interviewees from local pharmaceutical manufacturers with knowledge of the QA process, CEOs and members of the manufacturing association (Pakistan Pharmaceutical Manufacturing Association, PPMA);
3. academia, comprised of academics and public health researchers with experience and knowledge of local pharmaceutical policy and practice, and who could provide a more neutral perspective on the questions asked.

**Figure 1:**
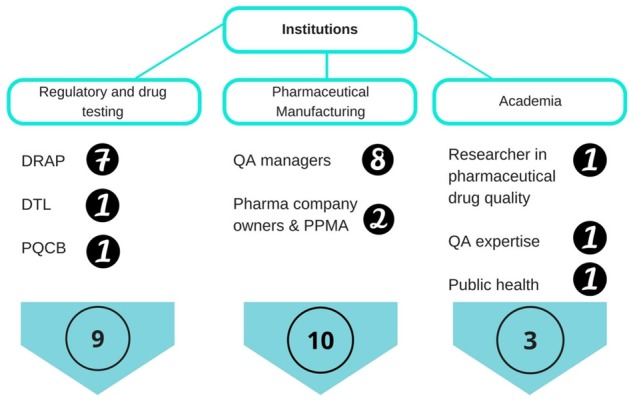
Groups of stakeholders and number of interviewees recruited for the study. DRAP, Drug Regulatory Authority of Pakistan; DTL, drug testing laboratory; PQCB, Punjab Quality Control Board; QA, quality assurance; PPMA, Pakistan Pharmaceutical Manufacturing Association.

The manufacturing group included both large- and small-scale companies. All companies produced for the domestic market and export. The list of potential participants was generated by two sampling techniques as no central register existed from which to recruit and invite participants. First, publicly available websites were used to identify potential interviewees. During this stage, 35 candidates were approached and 15 accepted the invitation to participate. Second, a snowballing strategy was used whereupon the 15 initially contacted interviewees suggested 12 additional interviewees to approach. From this sample, seven more interviewees were recruited, making it 22 interviewees in total. Of these, nine were from the regulatory group, 10 from the manufacturing group, and three from the academic group (Figure 1). The principal investigator (FT) contacted all participants through email and phone calls and conducted all the interviews. Prior to conducting the interviews, written consent was obtained from all participants after a verbal presentation of the project’s objectives and the procedures for managing the interview data. The principal investigator FT is a pharmacist with a MPhil in International Community Health. UG is a physician and researcher on health policy and global health governance, including R&D and access to medicines, with prior experience in qualitative methods and primarily supervised FT on the methodological approach. KM is a pharmacist with a master's degree in public health with extensive working experience from all areas of pharmaceutical policy, including the supply chain, access to medicines and developing a methodology to measure medicine prices. KM supervised FT on quality issues, including falsified medicines.

Seventeen interviews took place face to face at the workplaces of the interviewees (lasting 30–60 min), two were conducted via telephone (lasting 30 min), two via Skype (lasting 30–45 min) and one interviewee submitted the response in writing via email. During two interviews with the QA managers at the manufacturing site, their entire QA/QC teams were present (4 and 8 members). All interviews were conducted in Urdu.

The interview guide was informed by a review of the literature on the subject, which helped develop open-ended questions about experiences and perspectives that were considered relevant to help answer the research questions (Supplementary File S2). It was pretested with two experienced pharmacists (academic and industry) to assess the relevance of the questions and was modified after their feedback.

Audio recording of file names, transcriptions translated from Urdu to English, and field notes collected during fieldwork were stored in the researcher’s personal password-protected computer, in accordance with the procedures described in the approved application. Transcriptions and field notes were pseudonymized and the key linking audio recordings to the interviewees was kept in an encrypted folder in the computer.

### Document review

Document review was conducted to generate insights into the local pharmaceutical practices and the efforts to adhere to national and international standards. Moreover, insights gained from the interviews were compared with how local pharmaceutical regulations and practices were articulated in the policy documents. Regulatory and policy documents were collected from DRAP’s website, WHO and other agencies, and documents were also reviewed upon suggestions from the interviewees. The key documents reviewed were: Drugs Act 1976 of Pakistan—Schedule B, DRAP Act 2012 and SROs; PPMA annual report, 2017; WHO GMP guidelines (Annex 2, WHO Technical Report Series 986, 2014); Quality Assurance of Pharmaceuticals: A Compendium of Guidelines Vol 2, 2004; WHO report on Local Production of Pharmaceuticals and Related Technology Transfer in Developing Countries, 2011; and mainstream media reports (Supplementary File S3).

### Data analysis

The aims of the qualitative data analysis were: (1) to identify common themes from across the three main stakeholders; and (2) to identify contrasting experiences and views on the topics raised across the three main stakeholders. Together, these insights were interpreted to help explain the main barriers and enablers to implementing GMP in the local pharmaceutical industry, and its current QA system. The analytic strategy followed the steps for thematic analysis described by Braun and Clarke (2006). In addition, it was also inspired by the five-cycle process described by Yin, which follow similar steps to organizing and analyzing the data (Yin, 2015).

First, all the transcripts were read to get familiarized with the data, to identify immediate prominent findings and make initial notes. Second, qualitative coding was conducted whereupon a code was assigned to a single sentence, several sentences or larger segments expressed by the interviewee. A ‘code’ in this context can be understood as a concept or a short phrase that summarizes and captures the essence of a phenomenon described by the interviewee that are relevant for answering the research questions. The formation of these codes involved interpretation on part of the investigators. The list of codes was organized in Excel to help identify connections among them (e.g. whether the codes described similar or different phenomena of interest). Codes describing the similar phenomena were grouped together under overarching concepts and themes. Finally, to present the main findings, the identified themes were categorized under one of the three main areas explored by the study: (1) the current situation of QA incorporating GMP; (2) barriers to QA incorporating GMP implementation; (3) enablers to QA incorporating GMP implementation.

For document analysis, all relevant documents were gathered and read. To integrate insights from documents with the interview data, relevant data to compare the findings from the interview was highlighted and organized thematically in Excel.

### Ethics

The study obtained ethical clearance from the Norwegian Data Protection Official for Research on 05-08-2017 (Project no. 54772). Local ethical clearance was obtained on 01-11-2017 from Human Ethical Committee, University of the Punjab. Prior to consent, all the participants were informed that participation was voluntary, and that confidentiality would be maintained in publications. All participants consented to the study have been informed that participation was voluntary, and that confidentiality would be maintained in publications.

## Results

The thematic analysis of the qualitative data uncovered five major themes responding to our research questions.

### Coexistence of manufacturing facilities under different GMP standards and interpretations suggest an absence of harmonization in quality standards across the industry

Our findings indicated lack of harmonization in quality standards due to different GMP standards and interpretations being in place. The federal enforcement of GMP principles defined in law as Schedule B II under the Drug (Licensing, Registering and Advertising) Rules of 1976 (DRAP, 2019b) has been the responsibility of DRAP, which comprise federal drug inspectors and drug testing laboratories. Schedule B II, a set of minimum national standards, is mandatory for all the local pharmaceutical manufacturers. Our comparison of documents suggests that it has not been updated in accordance with WHO’s current GMP standards. One academic pointed out that Schedule B II has not been appropriately adapted to the local context and needed revision. In this regard, drug inspectors reported that revising the Act needed time. Hence, they attempt to enforce and the pharmaceutical industry aim to adhere to current WHO standards. They assess the manufacturing facilities based on conformance to current standards through an audit proforma in Schedule B II, which consists of questions about GMP interpreted for the most part from current WHO GMP guidelines. Moreover, they have sporadically proposed statutory notifications (SROs) and amendments to the Drug Act of 1976 to make the procedures easier for the manufacturers, marketers and sellers of drugs. Assurance of maintaining current GMP compliance and quality of the finished products, under the prescribed rules throughout the certified period of 3 years, is the responsibility of the facility.

In addition to WHO guidelines, according to the regulators, other international GMP standards developed by five key bodies such as ICH, EU, USFDA and Australia's Therapeutic Goods Administration (TGA) were also considered as references. Documents such as mainstream media reports revealed that only a few companies followed and more were looking into international GMP guidelines. A concise list of local companies had established facilities that contract manufacture for a variety of international companies as per international standards.

### Diverging experiences among stakeholders about the status and knowledge of GMP compliance in the local pharmaceutical industry

At the time of writing, no official report^1^ was found on DRAP’s website that provided local GMP enforcement and compliance data, but regulators expressed that the standards laid out by the Drug Act 1976 had been inadequately enforced in the past. However, with the substandard drug incident in Lahore, and creation of the DRAP Act of 2012, DRAP had taken steps towards making GMP conformance a legal requirement. This step was thought to protect the market from many local non-compliant companies. The dominant view among the regulators was that the local pharmaceutical sector over time had improved its adherence to WHO GMP guidelines, with many companies attempting to comply with other internationally accepted guidelines in order to remain competitive:

*I won’t say 100% of the local industry is aiming for GMP accreditation. The older ones are a bit reluctant; they need some time as it is impossible to upgrade conventional facilities. The point is, those who don’t want to improve, have no scope left. They will be left behind in the race* (Interviewee no. 19, regulator).

Interviewees from the manufacturing group stated that many companies were struggling to conform to the WHO GMP guidelines, reporting that a number of local manufacturing facilities in Lahore had closed. One industry interviewee estimated that the number of local companies with good GMP portfolios, high revenues and substantial resources were not >50. One QA manager of a well-reputed local manufacturer shared views on negligible international accreditation, expressing that ‘Quality needs validation. To date, Pakistan’s pharmaceutical industry’s international validation is limited’ (Interviewee no. 5, QA). This view is consistent with several media reports that have highlighted the lack of FDA approved facilities in Pakistan (Khan, 2016), limited number of WHO prequalified products (Abbasi, 2018) and that the regulatory authority is not a member of the Pharmaceutical Inspection Convention/Cooperation Scheme (PIC/S) (A Reporter, 2018). Interviewees among manufacturers also informed that most of the medicine exports were to low-income countries and not to better regulated markets.

Findings obtained from the question about GMP understanding revealed some ambiguity with respect to what substandard products were understood to be. One interviewee from the manufacturing group and one regulator stressed that a slight deviation in physical quality standards should not mean that the product is out of specifications. For example, one of these expressed:

*A little more friability or hardness, a slight change in colour, physical state or weight variation and the medicine is considered substandard; this doesn’t mean that the drug doesn’t have active ingredient* (Interviewee no. 16, regulator).

Moreover, these two interviewees stressed that an occasional mistake causing a batch to not meet requirements should be dealt with differently from systematic violations such as consistently having too little active ingredient. They stated that licensed providers that were not fully conforming to WHO GMP were at least complying with some basic manufacturing standards and were not making falsified medicines.

From the perspective of the stakeholders from academia, internationally accepted GMP compliance was considered inconsistent across the country. Only a handful of companies were considered to do exceptionally well in terms of meeting their regulatory obligations. When asked about the main achievements in GMP compliance, it was informed that no breakthroughs have been achieved in the last 10 years except for implementation of a Heating, Ventilation and Air-Conditioning system and initiation of stability studies. However, our study was not able to verify whether this statement reflected an accurate assessment of local manufacturers’ achievements with respect to GMP.

### Resource challenges associated with raw materials, infrastructure, technology and technically skilled personnel present barriers to sustainable compliance with and enforcement of quality standards

Industry interviewees argued that a substantial amount of continuous investment is needed to upgrade a pharmaceutical facility to meet WHO GMP and QA requirements. In the local sector, finances were limited for the introduction of new technology and upgraded machinery. A representative from academia described this quality vs cost issue as:

*everybody wants to implement GMP, but money is a barrier. Manufacturers step back when they see the need to invest millions to buy equipment. So, they try finding the middle ground and compromise on this* (Interviewee no. 1, academia).

Heavy duties on imported APIs and other raw materials were identified as another barrier by the manufacturers. It was emphasized that most of the local manufacturers import APIs from India and China. Very few were undertaking basic or semi-basic manufacturing as manufacturing raw materials locally was considered too costly. In addition to the operational costs, shipping and transporting APIs were considered resource-intensive, especially for companies away from the ports of Karachi. Apart from expenses associated with the raw materials, affordability of reference standards was another concern for small facilities. An academic with experience of GMP auditing informed:

*Local small companies in Lahore (Punjab) cannot afford expensive reference standards. This is a huge financial burden as these companies have few sections; sometimes just one or two. Purchasing the primary standards is a big deal for them* (Interviewee no. 3, academia).

General infrastructural issues were also described to be costly for manufacturers. For example, interviewees from the manufacturers reported that frequent electricity breakdowns were a big issue for some manufacturing facilities, except for those that have their own electricity supply, such as Sundar industrial estate in Lahore. Everyday power supply shortages make temperature and humidity conditions fall on either side of the allowed ranges, thereby affecting the products. As a result, owners have to invest in heavy-duty generators—an additional cost to their basic operational expenses—to keep the processes up and running and to avoid undesired production terminations.

Limited financial resources were also a barrier to embracing new technology in some facilities as well as for regulators. Among some local manufacturers, there was limited or no computerized system for e-record maintenance and no internet facility, which delayed updates on new policies and fluctuating market dynamics, and increased vulnerability to human errors. Four QA managers pointed out the administrative challenges with physical documentation of the batches, which sometimes lead to errors in streamlining the batch processing and packaging. On the regulatory side, DRAP officials from the regional office reported that so far, all the records were mainly manual documents.

Finally, a shortage of technically skilled personnel was identified as a significant bottleneck. Among the manufacturers, interviewees reported that technical skills were in shortage and most companies had poorly trained staff. Training and knowledge were primarily only at the managerial level. Many local manufacturers could not afford to arrange advanced-level training sessions for their employees or recruit qualified pharmacists, chemists and technicians and business managers. From the regulatory side, it was informed that even though the field force was increased in 2017, the number of drug inspectors were still insufficient. Accordingly, the market authorizations faced considerable delay. Regarding regulatory expertise, an academician highlighted that professionals working in the drug testing laboratories had limited knowledge regarding use of analytical and testing instruments and equipment.

### Many profit-oriented manufacturers yet to institutionalize a mindset promoting quality and safety

WHO GMP guidelines emphasize that manufacturers and their top management should establish a quality system within their entities (WHO, 2014a). However, QA managers and regulators highlighted the lack of an institutional quality mindset as a major barrier. Regulators argued that some pharmaceutical investors showed limited interest in strengthening their QA systems, but rather promoted a profit-oriented approach at the expense of the quality needed to manufacture essential medicines. For instance, a DRAP official described the pharmaceutical investors as businessmen who ‘want to opt for low risk investment options’ (Interviewee no. 22, regulator). In relation to this, an academic stated:

*Investors are not ready to comply with GMP principles to avoid any expenditure on their part and always try to comply at a level where no money has to be paid. For this, they even utilize political pressures, if required* (Interviewee no. 2, academia).

Most owners perceive GMP as a one-time process and not as a continuously evolving process that requires up-to-date technologies and procedures. Most QA managers expressed that their departments were progressively playing a crucial role in terms of assuring the quality and safety of the products. However, some managers pointed out that the quality aspect was generally not recognized and at times not understood by the top management and the working staff. In some facilities, owners viewed QA as the job of the QA management alone. There was a lack of authority and ownership of QA and the owners often interfered. One QA interviewee stated that a QA department could only work independently when it was given authority over batch release. This interviewee stated that owners would not allow the QA to stop the production as it was a ‘nightmare’, i.e. a huge economic loss to them. In relation to validity of records and procedures, a QA manager expressed that process validations or revalidations were not taken seriously at times.

Overall, interviewees from the manufacturing group described experiences confirming the notion of difficulty in maintaining an institutional quality mindset. Interviewees from DRAP and academia reinforced this notion, expressing that acceptability and understanding of the impact of QA was currently lacking among local industry. A DRAP senior official claimed:

*There is limited awareness regarding importance of drug quality and safety and less understanding regarding the regulations and rules. Stakeholders should understand that these issues are important and if they want to develop the local industry then an understanding of the rules is very pivotal* (Interviewee no. 20, regulator).

Moreover, one academic interviewee pointed out:

*Irresponsible attitude of regulators and stakeholders regarding recognition of their responsibility is a barrier; I mean, they are responsible for the health of people, thousands of lives will be at stake if they make one mistake* (Interviewee no. 2, academia).

### Establishment of DRAP represented a federal-level institutional change that has promoted QA through inspections and guidance, but suffer from insufficient resources to meet the technical and regulatory needs of a growing number of manufacturers and products

A strong regulatory system is necessary for the enforcement of QA of medicines. Contrasting views and experiences about the establishment of DRAP were expressed among the interviewees from the three groups. The document analysis of statutory notifications and the interviews with the regulators indicated that the presence of DRAP had triggered QA activity among manufacturers. Newsletters available on DRAP’s website, newspapers and interviews with the regulators together indicated that DRAP has taken many regulatory measures, including market surveys, strict check campaigns against SF drugs, and raids to sales outlets and manufacturing facilities. To promote QA, it was aiming towards international best practices for harmonization of regulatory functions. DRAP had mandated an independent head of QA to have a pharmacy degree with significant experience in quality control and testing of drugs. Regulators informed that the regulations concerning QA were more stringent than before. Previously, many small manufacturers would hide the quality errors fearing that inspectors would investigate and close their facility. Now, internal audits and self-inspection had been added in the GMP report and many companies generated their self-inspection reports indicating the cause of quality failure and subsequent corrective efforts. Moreover, they were encouraged to develop corrective and preventive actions (Motschman and Moore, 1999) and perform root cause analysis. At the same time, regulators seemed to give manufacturers time to adapt and make improvements to meet WHO standards. One DRAP official stated that fair time was given to facilities to shift according to the current standards:

*A company that has just begun, we can’t expect it to reach the level of a top pharma company, or a multinational. It’s not possible. We must support our industry. For that, keeping practicality is crucial, we do give some room to such facilities where quality isn’t compromised but some improvements are required* (Interviewee no. 17, regulator).

In stark contrast to the above, interviewees from academia expressed that insufficient regulatory capacity of DRAP was a barrier to the implementation of GMP and QA. Basic legislation and regulation of quality standards and enforcement capacity of DRAP was described to be weak. One academic highlighted that DRAP’s establishment in 2012 was not a watershed event. The interviewee pinpointed a dearth of neutrality and conflict of interest among DRAP officials. No official annual reports were generated by DRAP on its website that stated how the regulators or the industry were performing. Furthermore, no objective information was available in modified form for public accountability.

Communication challenges between DRAP and provincial drug control units were highlighted by two manufacturers. According to the rule, the provincial governments are responsible for the regulation of drug sale only (DRAP, 2019a, 2019b). The manufacturers interviewed informed that the miscommunication had led to mistrust and tension among the manufacturers:

*a DRAP inspector would suggest that this door should be here and later a provincial inspector would overrule it. These authorities do not communicate with each other to develop a consensus which ends up discouraging the manufacturers as they have to deal with two bodies* (Interviewee no. 13, manufacturer).

Another set of shortcomings that were highlighted regarding the market authorizations were backlogs and no clear timelines. Adding to the procedural deficiencies, it was informed that bioequivalence studies were not required for product registration. In addition, flaws in compliance testing by the DRAP such as avoiding assessment of the quality of APIs, and their specifications and standards before registration were highlighted by two academic interviewees:

*Assessment and approval of specifications and standards is not being carried out and granted in an appropriate and lawful manner. It is major lacuna being faced by regulators at different levels, e.g. if a firm’s medicines are declared substandard, it would approach the courts and would get the relief in its favour after claiming the specifications of its interest. All this is because specifications are loosely covered under current set of regulations in Pakistan and ignored at the time of registration* (Interviewee no. 3, academia).

From the manufacturers’ perspective, three QA managers pointed out the vague checks on validations, changes to specifications and manufacturing and product development processes by the drug inspectors. Two QA managers reported that the inspectors only infrequently made unannounced visits. Usually, the inspections were planned, which gave some facilities time to minimize their production and focus on cleanliness on the inspection day. Accordingly, the manufacturers’ experience seemed to indicate that strengthening the capacity of regulatory authorities is needed.

In recent months, DRAP in partnership with Promoting the Quality of Medicines (PQM) programme has made significant developments to strengthen its QA system and to achieve WHO Maturity Level (ML) III^2^ based on global benchmarking tool (GBT), which include Pakistan Drug Testing and Research Centre (PDTRC) accreditation with WHO, membership of international monitoring system for pharmacovigilance, and adoption of Common Technical Document (CTD) format for market authorization (PQM, 2018).

## Discussion

This study highlights several barriers and enablers in implementing and enforcing QA and GMP standards in the local pharmaceutical sector of Pakistan. Insufficient financial resources, limited technologies, and infrastructural, administrative and technical challenges are major barriers to sustainably implementing, enforcing and adhering to quality standards. Previous studies have reported similar barriers (WHO, 1999; Brhlikova *et al.*, 2015; WHO, 2018b). A recent review by Roth *et al.* (2018) identified similar challenges among national regulatory authorities in LMICs, which restricted access to quality-assured medical products. Also, coexistence of manufacturing facilities under different GMP standards and interpretations points towards the long-standing challenge of global harmonization of quality standards and regulatory supervision.

In this study, one major barrier to QA and GMP compliance was found to be the difficulty of endorsing a culture of quality mindset among profit-oriented local manufacturers in a setting with a rapidly growing economy and low regulatory capacity. WHO’s GMP guidance encourages the senior management of pharmaceutical companies to establish a quality principle with ‘a comprehensively designed and correctly implemented system of QA incorporating GMP and quality control’ to produce quality medicines (WHO, 2007; WHO Drug Information, 2017). A previous review of GMP for vaccine production in LMICs identified the top management to not fully understand the significance of QA, with senior managers considering these measures an additional expense (Milstien *et al.*, 2009). Another study highlighted the lack of institutional commitment to invest in QA as a factor hindering the improvement of the quality of medicines for end users (Nebot Giralt *et al.*, 2017). A previous study in Pakistan has mentioned the reluctance of pharmaceutical investors to invest in additional tests such as stability and validation studies when applying for drug registration (Babar *et al.*, 2016). At a global level, a study stated that pharmaceutical manufacturers prefer to stick to the ‘tried and tested’ systems and for them to embrace innovative approaches is hard (Plumb, 2005). Hence, changing an established mindset seems to be difficult. Interestingly, in addition to perspectives from regulators and academia, our study supplements these previous findings with perspectives from within the pharmaceutical industry. Many of these interviewees provided candid assessments regarding this issue.

It should be noted that the challenge of establishing a quality culture is exacerbated by limited resources, both human and financial. Human resources are critical to implement and oversee essential steps in the QA process such as process validation and enforcement of regulations. WHO GMP guidelines highlight qualified personnel as indispensable to ensure optimal quality (WHO, 2014a). Our study identified limited technical capacity and qualified personnel to be major issues both within local manufacturers as well as within DRAP. Similar findings have been identified in other contexts. For example, a study on the impact of GMP on the local pharmaceutical sector of Nepal identified regulatory and technical incapacity of the manufacturers and the regulators as one of the many barriers in implementing GMP (Brhlikova *et al.*, 2015). In prior literature, insufficient financial resources have been recognized as an underlying factor limiting the implementation of a fully functional regulatory system and a completely adherent pharmaceutical industry (Caudron *et al.*, 2008; Vian et al., 2017; Roth *et al.*, 2018). Complying with GMP and assuring quality is an expensive long term and continuously evolving process and requires establishment and maintenance of a QA system. In a highly competitive market where regulatory capacity to oversee and enforce regulations are limited, companies might be tempted to skip certain steps, thereby risking the quality of their products (Milstien *et al.*, 2009; Anyakora *et al.*, 2017).

Regulatory capacity in LMICs has been reported to be inadequate (Roth *et al.*, 2018). Pakistan’s drug regulatory system and DRAP have previously been criticized for their institutional deficiencies and for failing to make an impact on the pharmaceutical landscape of the country (Nishtar, 2011; Rashid, 2015; Daily times monitor, 2017). Among the major issues have been that bioequivalence studies of generic medicines have not been a mandatory part of marketing approval (Babar *et al.*, 2016). Babar *et al.* (2016) proposed a range of technical requirements for issuing marketing authorizations for medicines in Pakistan, including bioequivalence studies of already registered generic drugs. For long, facilities for conducting bioequivalence studies were lacking in Pakistan (Hasan, 2013; Junaidi, 2018). This has improved in recent times with the establishment of a WHO prequalified PDTRC (PQM, 2018). In our study it was reported that due to issuance of stricter policies against substandard medicines, local companies that are unable to follow international standards are leaving the pharmaceutical sector and blaming the authorities for not being more supportive. In principle, patients are unlikely to benefit from too many companies competing for a small market share. Enforcing QA ensures that patients receive safe and effective quality medicines and builds trust in public health. Ultimately, beyond soft loans and reduction of taxes and tariffs, governments play a limited role in keeping the pharmaceutical business alive. Our study identified measures taken by DRAP to assure quality of locally produced medicines, such as counselling and giving local companies fair time, enforcing an independent QA system and encouraging companies to be above-board, and heading to achieve ML III. Yet the lack of transparency and accountability were highlighted as particularly important shortcomings by academia. These are factors that the WHO Global Surveillance and Monitoring System report 2017 identified to be important for effective management of the production and supply of medical products (WHO, 2018b). Supporters have emphasized that DRAP is in its early stages and that it takes time to set up its institutions and strengthen its capacity. Globally, national regulatory institutions such as DRAP together with other public health institutions play a crucial role in holding pharmaceutical companies accountable for ensuring the safety of their products when bringing these to market.

At the international level, the WHO Regulatory Systems Strengthening programme has since 2014 worked on developing a unified tool for the benchmarking of national regulatory systems for medicines and vaccines, with a final GBT expected to be finalized in 2019 (Azatyan, 2017). DRAP is prompting an institutional mindset for quality by aspiring to upgrade its ML to III, and by educating and facilitating the pharmaceutical industry to enhance its compliance with standards and technical capacity (Azatyan, 2017; Drug Regulatory Authority of Pakistan, 2018). Over time, DRAP should aim towards ML IV. This would require increased public investment and strengthening of its own institutional capacity building. To improve efficiency and enhance quality, there are opportunities for participation in regional and international information-sharing to increase regulatory capacity, such as through the WHO collaborative procedure, which Pakistan joined in 2018 (Azatyan, 2017; WHO, 2018a, 2019). It might be worthwhile looking into establishing information sharing through an online platform to facilitate and enhance communication between manufacturers and regulators in order to improve QA documentation and record systems. Here, professional bodies with members from both industry and regulators can voluntarily share successful interventions that play a strong role in encouraging, developing and training in QA, incorporating GMP.

Another international mechanism that could motivate greater investments in QA among local manufacturers is increasing awareness of receiving WHO prequalification for their products, which would enable them to bid for international tenders, receive faster regulatory approval and increase exports (WHO, 2013b, 2014b). The Karachi-based company Getz Pharma became the first manufacturer from Pakistan to obtain WHO prequalification for a drug, namely moxifloxacin—an antibiotic in the fluoroquinolone class that is on WHO’s list of essential medicines (Abbasi, 2018). It is worth exploring in the future whether this milestone has motivated other companies to aim for similar standards, and DRAP’s activities to promote such efforts.

### Limitations and methodological considerations

The data collected for this study was sourced from a limited number of companies from one geographic location (Lahore). Accordingly, the findings of this study might not necessarily represent the experiences of companies in other parts of Pakistan or the local pharmaceutical landscape more generally. The companies that were unreachable might differ in important aspects from those we reached, e.g. that we might have only been informed by the experience of those performing reasonably well. The inclusion of interviewees from manufacturers not only presents a source for valuable insights, but also carries the risk of the study being informed by data with social desirability bias—that interviewees report experiences in a way that place the manufacturers in a favourable light (Lewis-Beck *et al.*, 2004). However, our interpretation is that most interviewees presented a candid assessment, including reporting negative experiences and admitting to weaknesses. Regarding the manufacturers represented among the interviewees, it was not investigated what range and type of drugs they were producing and whether they were delivering to the government hospitals. However, during the interviews, they were enquired about the range and essentiality of their products. All the companies were producing medicines for the public sector, in addition to their private consumers.

## Conclusion

The local manufacturers and regulators of medicines in Pakistan face, despite recent institutional improvements, a set of challenges in terms of implementing a fully functional QA system incorporating current WHO GMP guidelines. The challenges include limited financial resources, inadequately trained personnel, a dearth of quality-oriented institutional mindset, limited infrastructural and technological advancements, and insufficient capacity at the drug regulatory authority. A profit-oriented mindset among manufacturers without taking the responsibility of implementing a QA system of international standards seriously can have severe public health implications, particularly when regulatory capacity is low. The challenges observed in Pakistan are shared with other LMICs with growing local pharmaceutical production, and the problem of substandard medicines can spread between countries. Accordingly, there is a need to enhance commitment to international collaborative mechanisms, such as those lead by WHO, on this issue.

## Supplementary Material

czz054_Supplementary_Files

## Footnotes

1 During the revision of this article, we identified that DRAP on January 18, 2019 released a press report in Urdu on its Facebook page that reports number of registered companies, companies given GMP certifications, samples tested, inspections made, etc.

2 WHO Regulatory Systems Strengthening programme aims to classify regulatory functions using a harmonized and systematic GBT based on four maturity levels (MLs); ML1 and ML2 implies that the regulatory system performs partially, and ML3 and ML4 implies that it is a WHO—listed institution, “operating at advanced level of performance and continuous requirement” (Azatyan, 2017).

## References

[czz054-B1] A Reporter. 2018 Drap should adopt automated system for drug inspection. *Dawn* https://www.dawn.com/news/1406657, accessed 5 January 2019.

[czz054-B2] Aamir M, Zaman K. 2011 Review of Pakistan pharmaceutical industry: SWOT analysis. International Journal of Business Information Technology 1: 114–7.

[czz054-B3] Abbasi W. 2018 *WHO Accredits First-Ever Pak Drug* [Online]. The News. https://www.thenews.com.pk/print/278741-who-accredits-first-ever-pak-drug, accessed 1 April 2018.

[czz054-B4] Anyakora C, Ekwunife O, Alozie F et al 2017 Cost benefit of investment on quality in pharmaceutical manufacturing: WHO GMP pre- and post-certification of a Nigerian pharmaceutical manufacturer. BMC Health Services Research 17: 665. 28923044 10.1186/s12913-017-2610-8 PMC5604295

[czz054-B5] Atif M, Ahmad M, Saleem Q et al 2017 Pharmaceutical policy in pakistan In: Babar Z-U-D (ed). Pharmaceutical Policy in Countries with Developing Healthcare Systems. Cham: Springer International Publishing, pp. 25–44.

[czz054-B6] Azatyan S. 2017 *WHO PQ Collaborative Registration Procedure and SRA Collaborative Procedure* https://www.who.int/pq-vector-control/resources/orient_crp.pdf?ua=1, accessed 3 March 2019.

[czz054-B7] Babar A, Khan B, Godman B, Hussain S, Mahmood S, Aqeel T. 2016 Assessment of active pharmaceutical ingredients in drug registration procedures in Pakistan: implications for the future. Generics and Biosimilars Initiative Journal 5: 156–63.

[czz054-B8] Bassat Q, Tanner M, Guerin PJ, Stricker K, Hamed K. 2016 Combating poor-quality anti-malarial medicines: a call to action. Malaria Journal 15: 302. 27251199 10.1186/s12936-016-1357-8 PMC4888506

[czz054-B9] Braun V, Clarke V. 2006 Using thematic analysis in psychology. Qualitative Research in Psychology 3: 77–101.

[czz054-B10] Brhlikova P, Harper I, Subedi M et al 2015 Aid conditionalities, international good manufacturing practice standards and local production rights: a case study of local production in Nepal. Globalization and Health 11: 25. 26072308 10.1186/s12992-015-0110-3 PMC4470019

[czz054-B12] Caudron J, Ford N, Henkens M et al 2008 Substandard medicines in resource‐poor settings: a problem that can no longer be ignored. Tropical Medicine & International Health 13 1062–72. 18631318 10.1111/j.1365-3156.2008.02106.x

[czz054-B13] Chaudhry A. 2013 *WHO Says Drug Caused PIC Deaths* Dawn. https://www.dawn.com/news/797093, accessed 12 March 2019.

[czz054-B14] Chin R, Lee BY (eds). 2008 Chapter 2—ethical, legal, and regulatory issues. In: Principles and Practice of Clinical Trial Medicine. New York, NY: Academic Press, pp. 17–39.

[czz054-B16] Daily Times Monitor. 2017 Problems faced by Pakistan's pharma industry. *Daily Times* https://dailytimes.com.pk/36555/problems-faced-by-pakistans-pharma-industry/, accessed 1 May 2018.

[czz054-B17] Drug Regulatory Authority of Pakistan (DRAP). 2018 *CEO DRAP Stressed on the Need of Harmonization of Standards on Regulations of Medicines and Vaccines among Regulatory Authorities of OIC Countries.* https://www.dra.gov.pk/docs/media/PRDRAP28-11-18.pdf, accessed 21 March 2019.

[czz054-B18] Drug Regulatory Authority of Pakistan (DRAP). 2019a *Drug Regulatory Authority of Pakistan Act, 2012* https://www.dra.gov.pk/docs/DRAP%20Act.pdf, accessed 4 April 2018.

[czz054-B19] Drug Regulatory Authority of Pakistan (DRAP). 2019b *The Drugs Act, 1976* https://www.dra.gov.pk/docs/TheDrugsAct1976111115F.pdf, accessed 4 April 2018.

[czz054-B20] Drug Regulatory Authority of Pakistan (DRAP) Press Release. 2019 *Facebook Page* https://www.facebook.com/OfficialDRAP/photos/pcb.599777147120351/599777007120365/? type=3&theater, accessed 1 May 2019.

[czz054-B21] European Commission (EC). 2018 *EudraLex—Volume 4—Good Manufacturing Practice (GMP) Guidelines. Part I Basic Requirements for Medicinal Products* https://ec.europa.eu/health/documents/eudralex/vol-4_en, accessed 2 May 2018.

[czz054-B22] Geyer ARC, Sousa VD, Silveira D. 2018 Quality of medicines: deficiencies found by Brazilian Health Regulatory Agency (ANVISA) on good manufacturing practices international inspections. PLoS One 13: e0202084. 30089162 10.1371/journal.pone.0202084 PMC6082550

[czz054-B23] Global Antibiotic Resistance Partnership (GARP). 2018 *Situation Analysis Report on Antimicrobial Resistance in Pakistan: Findings and recommendations for Antibiotic Use and Resistance.* https://cddep.org/publications/garp-pakistan-situation-analysis/, accessed 2 May 2019.

[czz054-B24] Hasan S. 2013 Prospects of drug bioequivalence studies in Pakistan. Journal of the Dow University of Health Sciences 6 http://jduhs.com/index.php/jduhs/article/view/16, accessed 3 March 2019.

[czz054-B15] Institute of Medicine. 2013 Countering the Problem of Falsified and Substandard Drugs. Washington, DC: The National Academies Press. 24872973

[czz054-B25] International Conference on Harmonization (ICH). 2019 *Quality Guidelines* https://www.ich.org/products/guidelines/quality/article/quality-guidelines.html, accessed 26 February 2019.

[czz054-B26] Johnston A, Holt DW. 2014 Substandard drugs: a potential crisis for public health. British Journal of Clinical Pharmacology 78: 218–43. 24286459 10.1111/bcp.12298 PMC4137817

[czz054-B27] Junaidi I. 2018 Committee formed to look into establishment of bioequivalence lab. *Dawn* https://www.dawn.com/news/1395931, accessed 4 March 2019.

[czz054-B28] Kelesidis T, Falagas ME. 2015 Substandard/counterfeit antimicrobial drugs. Clinical Microbiology Reviews 28: 443–64. 25788516 10.1128/CMR.00072-14 PMC4402958

[czz054-B29] Khan AS. 2016 No FDA-approved pharmaceutical plant in Pakistan: SBP. *Dawn*, November 24.

[czz054-B30] Lewis-Beck MS, Bryman A, Liao TF (eds). 2004 The SAGE Encyclopedia of Social Science Research Methods. Thousand Oaks, CA: SAGE Publications.

[czz054-B31] Milstien J, Costa A, Jadhav S, Dhere R. 2009 Reaching international GMP standards for vaccine production: challenges for developing countries. Expert Review of Vaccines 8: 559–66. 19397413 10.1586/erv.09.23

[czz054-B32] Motschman TL, Moore SB. 1999 Corrective and preventive action. Transfusion Science 21: 163–78. 10747525 10.1016/s0955-3886(99)00088-0

[czz054-B33] Nebot Giralt A, Schiavetti B, Meessen B et al 2017 Quality assurance of medicines supplied to low-income and middle-income countries: poor products in shiny boxes? BMJ Global Health 2: e000172. 10.1136/bmjgh-2016-000172 PMC5435257 28589013

[czz054-B34] Newton PN, Green MD, Fernández FM. 2010 Impact of poor-quality medicines in the ‘developing’ world. Trends in Pharmacological Sciences 31: 99–101. 20117849 10.1016/j.tips.2009.11.005 PMC2845817

[czz054-B35] Nishtar S. 2011 Drug regulation and beyond. *The New International* http://www.heartfile.org/wp-content/uploads/2014/12/101_DRUG_REGULATION_AND_BEYOND.pdf, accessed 4 April 2018.

[czz054-B36] Nishtar S. 2013 Handover Papers: Towards Improving Governance. http://sanianishtar.info/pdfs/HOP-Compendium_Final.pdf, accessed 2 March 2019.

[czz054-B37] Nishtar S, Mehboob AB. 2011 Pakistan prepares to abolish Ministry of Health. Lancet 378: 648–9. 21549420 10.1016/S0140-6736(11)60606-5

[czz054-B38] Nwokike J, Clark A, Nguyen PP. 2018 Medicines quality assurance to fight antimicrobial resistance. Bulletin of the World Health Organization 96: 135–7. 29403117 10.2471/BLT.17.199562 PMC5791778

[czz054-B39] Patel KT, Chotai NP. 2011 Documentation and records: harmonized GMP requirements. Journal of Young Pharmacists 3: 138–50. 21731360 10.4103/0975-1483.80303 PMC3122044

[czz054-B40] Patton MQ. 2002 Qualitative Research & Evaluation Methods*.* Thousand Oaks, CA: Sage Publications.

[czz054-B41] Petersen A, Held N, Heide L; Difäm-EPN Minilab Survey Group. 2017 Surveillance for falsified and substandard medicines in Africa and Asia by local organizations using the low-cost GPHF Minilab. PLoS One 12: e0184165. 28877208 10.1371/journal.pone.0184165 PMC5587284

[czz054-B65] Pezzola A, Sweet CM 2016 Global pharmaceutical regulation: the challenge of integration for developing states. Globalization and health 12: 85. 27998293 10.1186/s12992-016-0208-2 PMC5175325

[czz054-B42] Plumb K. 2005 Continuous processing in the pharmaceutical industry: changing the mind set. Chemical Engineering Research and Design 83: 730–8.

[czz054-B43] Policy Research Institute of Market Economy. 2017 *Pakistan’s Pharmaceutical Industry* http://www.ppma.org.pk/wp-content/uploads/2017/09/Final-Report-Pharma-Industry_August-10.pdf, accessed 3 March 2019.

[czz054-B44] Promoting the Quality of Medicines (PQM) Program. 2018 *FY 2018 Fourth Quarter Report* https://www.usp-pqm.org/sites/default/files/pqms/f18-q4-report.pdf, accessed 3 May 2019.

[czz054-B45] Rashid H. 2015 Impact of the drug regulatory authority Pakistan: an evaluation. New Visions for Public Affairs 7: 50–61.

[czz054-B46] Roth L, Bempong D, Babigumira JB et al 2018 Expanding global access to essential medicines: investment priorities for sustainably strengthening medical product regulatory systems. Globalization and Health 14: 102. 30382856 10.1186/s12992-018-0421-2 PMC6211488

[czz054-B47] Statista. 2019 *Global Pharmaceutical Industry—Statistics & Facts.* https://www.statista.com/topics/1764/global-pharmaceutical-industry/, accessed 3 January 2019.

[czz054-B48] U.S. Food and Drug Administration. 2018 *Current Good Manufacturing Practice (CGMP) Regulations* U.S. Food and Drug Administration. https://www.fda.gov/Drugs/DevelopmentApprovalProcess/Manufacturing/ucm090016.htm, accessed 10 February 2019.

[czz054-B49] UNDESA. 2018 *Sustainable Development Goal 3.* Division for sustainable development, United Nations Department of Economic and Social Affairs. https://sustainabledevelopment.un.org/sdg3, accessed 3 January 2019.

[czz054-B50] Vian T, Kohler JC, Forte G, Dimancesco D. 2017 Promoting transparency, accountability, and access through a multi-stakeholder initiative: lessons from the medicines transparency alliance. Journal of Pharmaceutical Policy and Practice 10: 18. 28588896 10.1186/s40545-017-0106-x PMC5457587

[czz054-B51] World Health Organisation (WHO). 1999 *Counterfeit Drugs. Guidelines for the Development of Measures to Combat Counterfeit Drugs* http://apps.who.int/medicinedocs/en/d/Jh1456e/#Jh1456e, accessed 10 April 2018.

[czz054-B52] World Health Organisation (WHO). 2007 Quality Assurance of Pharmaceuticals: A Compendium of Guidelines and Related Materials. WHO. Geneva. http://apps.who.int/medicinedocs/documents/s14136e/s14136e.pdf, accessed 2 March 2018.

[czz054-B53] World Health Organisation (WHO). 2010 Monitoring the Building Blocks of Health Systems: A Handbook of Indicators and Their Measurement Strategies. World Health Organization Geneva. https://www.who.int/healthinfo/systems/WHO_MBHSS_2010_full_web.pdf, accessed 7 December 2018.

[czz054-B54] World Health Organisation (WHO). 2013a *Deadly Medicines Contamination in Pakistan.* http://www.who.int/features/2013/pakistan_medicine_safety/en/, accessed 12 February 2018.

[czz054-B55] World Health Organisation (WHO). 2013b Investing in WHO Prequalification of Finished Pharmaceutical Products. Information for manufacturers. WHO Drug Information Geneva. https://extranet.who.int/prequal/sites/default/files/documents/WHO_Prequalification_WHY_2.pdf, accessed 12 January 2019.

[czz054-B56] World Health Organisation (WHO). 2014a Annex 2, WHO Good Manufacturing Practices for Pharmaceutical Products: Main Principles. 48 ed Switzerland: WHO headquarters.

[czz054-B57] World Health Organisation (WHO). 2014b WHO Prequalification. Building Quality-Assured Manufacturing Capacity in Nigeria. WHO Drug Information Geneva. http://apps.who.int/medicinedocs/documents/s21721en/s21721en.pdf, accessed 5 December 2018.

[czz054-B58] World Health Organisation (WHO). 2018a Pakistan joins the collaborative registration procedure. https://extranet.who.int/prequal/news/pakistan-joins-collaborative-registration-procedure, accessed 3 April 2019.

[czz054-B59] World Health Organisation (WHO). 2018b *WHO Global Surveillance and Monitoring System for Substandard and Falsified Medical Products*. WHO/EMP/RHT/2017.01. WHO 2017. Geneva, Switzerland.

[czz054-B60] World Health Organisation (WHO). 2018c *Medicines Quality Assurance* http://www.who.int/medicines/areas/quality_safety/quality_assurance/en/, accessed 17 April 2018.

[czz054-B61] World Health Organisation (WHO). 2018d *Substandard and Falsified Medical Products* http://www.who.int/mediacentre/factsheets/fs275/en/, accessed 18 April 2018.

[czz054-B62] World Health Organisation (WHO). 2019 *Accelerated Registration of Prequalified FPPs.* https://extranet.who.int/prequal/content/collaborative-registration-faster-registration, accessed 10 April 10 2019.

[czz054-B63] World Health Organisation (WHO) Drug Information. 2017 Norms and standards—70 years of who standards on medicines quality. WHO Drug Information 31: 15–26.

[czz054-B64] Yin RK. 2015 Qualitative Research from Start to Finish. New York: Guilford Publications.

