# Supplementary material for: Institutional barriers and enablers to implementing and complying with internationally accepted quality standards in the local pharmaceutical industry of Pakistan: a qualitative study
Source: Health Policy Plan. 2019 Jul 13;34(6):440–9. doi: 10.1093/heapol/czz054 (PMC6736431; doi:10.1093/heapol/czz054)
Supplement: czz054_Supplementary_Files [file czz054_supplementary_files.zip › czz054-suppl_data/czz054_supplemnetary file S2.docx]

**INTERVIEW GUIDE FOR THE IN-DEPTH INTERVIEWS**

Questions to manufacturing group

1. Could you describe the current situation of GMP compliance and quality assurance of drugs in the local pharmaceutical setup?
2. What kind of products do you manufacture?
3. In your point of view, what are the key barriers to GMP compliance? Why?
4. How do you and your staff perceive GMP?

Questions to the regulatory group

1. How good manufacturing practice (GMP) regulations have been implemented locally and how these impact local medicines production?
2. It is known from some studies that products produced locally in the developing countries are usually of low quality what do you think about this in Pakistan’s context?
3. How are the aspects of quality of medicines assessed by DRAP?
4. In your point of view, what do you think are the main challenges in the implementation and enforcement of international quality assurance regulations in Pakistan? Which are the main challenges you have faced in the past/currently face in terms of QA inspection? Why?
5. What are the main achievements/improvements made in the process of quality assurance of medicines in Pakistan?

Questions to the academic group

1. Could you describe the current situation of GMP compliance and quality assurance of drugs in the local pharmaceutical setup?
2. In your opinion, what do you think are the main challenges in the implementation and enforcement of international quality assurance regulations in Pakistan? Why?
3. It is known from some studies that products produced locally in the developing countries are usually of low quality what do you think about this in Pakistan’s context?
4. How GMP and QA are perceived locally?
5. What are the main achievements/improvements made in the process of quality assurance of medicines in Pakistan?
