# Supplementary material for: Institutional barriers and enablers to implementing and complying with internationally accepted quality standards in the local pharmaceutical industry of Pakistan: a qualitative study
Source: Health Policy Plan. 2019 Jul 13;34(6):440–9. doi: 10.1093/heapol/czz054 (PMC6736431; doi:10.1093/heapol/czz054)
Supplement: czz054_Supplementary_Files [file czz054_supplementary_files.zip › czz054-suppl_data/czz054_supplementary file S3.docx]

**Mainstream media reports**

**Dawn**

1. ‘Drap should adopt automated system for drug inspection’. Available at: <https://www.dawn.com/news/1406657>

2. ‘WHO says drug caused PIC deaths’. Available at: <https://www.dawn.com/news/797093>

3. ‘Substandard drugs’. Available at: <https://www.dawn.com/news/1302324>

4. Enumerating Pakistan. Available at: <https://www.dawn.com/news/1354458>

5. ‘Committee formed to look into establishment of bioequivalence lab’. Available at: <https://www.dawn.com/news/1395931>

6. ‘No FDA-approved pharmaceutical plant in Pakistan: SBP’. Available at: <https://www.dawn.com/news/1298209>

7. ‘Drap asks nine firms to recall ‘contaminated medicines’ used to treat high blood pressure’. Available at: <https://www.dawn.com/news/1420369>

8. ‘Health-sector turnaround’. Available at: <https://www.dawn.com/news/1436081>

9. ‘Patients can import unregistered drugs: Drap’. Available at: <https://www.dawn.com/news/1391505>

10. ‘Punjab govt asks DRAP to cancel licences of 10 pharma firms’. Available at: <https://www.dawn.com/news/1278735>

**The Express Tribune**

1. ‘PIC suo motu: With 117 dead, interim report to be submitted Feb 6’. Available at: <https://tribune.com.pk/story/329793/pic-suo-motu-interim-report-to-be-submitted-on-feb-6-death-toll-reaches-117/>

2. ‘Temperature sensitive: Improper handling of drugs now punishable offence’. Available at: <https://tribune.com.pk/story/1321006/temperature-sensitive-improper-handling-drugs-now-punishable-offence/>

**The News International**

1. ‘WHO accredits first-ever Pak drug’. Available at: <https://www.thenews.com.pk/print/278741-who-accredits-first-ever-pak-drug>

2. ‘DRAP to launch revised cardiac registry soon’. Available at: <https://www.thenews.com.pk/print/342269-drap-to-launch-revised-cardiac-registry-soon>

**Miscellaneous**

‘Pak pharma industry suffers from government’s shackles’. Available at: <https://profit.pakistantoday.com.pk/2018/02/06/pak-pharma-industry-suffers-from-governments-shackles/>
